# Isolation and Sequencing of the Y Chromosome in Mediterranean River Buffalo Using Laser Microdissection-Based NGS

**DOI:** 10.3390/genes17070740

**Published:** 2026-06-26

**Authors:** Alfredo Pauciullo, Neyrouz Letaief, Ugo Ala, Halina Černohorská, Svatava Kubičková, Miluše Vozdová, Angela Perucatti, Leopoldo Iannuzzi, Giustino Gaspa, Yi Zhang, Gianfranco Cosenza

**Affiliations:** 1 Department of Agricultural, Forest and Food Sciences, University of Torino, Largo Paolo Braccini 2, 10095 Grugliasco, Italy; neyrouz.letaief@unito.it (N.L.); giustino.gaspa@unito.it (G.G.); 2 Department of Veterinary Sciences, University of Torino, Largo Paolo Braccini 2, 10095 Grugliasco, Italy; ugo.ala@unito.it; 3 Veterinary Research Institute, Hudcova 296/70, 62100 Brno, Czech Republic; halina.cernohorska@vri.cz (H.Č.); svatava.kubickova@vri.cz (S.K.); miluse.vozdova@vri.cz (M.V.); 4 Institute for the Animal Production System in the Mediterranean Environment, National Research Council of Italy, Piazzale Enrico Fermi 1, 80055 Portici, Italy; angela.perucatti@cnr.it (A.P.); leopoldo.iannuzzi@ispaam.cnr.it (L.I.); 5 State Key Laboratory of Animal Biotech Breeding, National Engineering Laboratory for Animal Breeding, Key Laboratory of Animal Genetics, Breeding and Reproduction of Ministry of Agriculture and Rural Affairs, College of Animal Science and Technology, China Agricultural University, Beijing 100193, China; yizhang@cau.edu.cn; 6 Department of Agriculture, University of Napoli Federico II, Piazza Carlo di Borbone 1, 80055 Portici, Italy

**Keywords:** Mediterranean river buffalo, Y-chromosome, laser microdissection, fluorescence in situ hybridization, next-generation sequencing, male-specific region of the Y chromosome, pseudoautosomal region, multi-copy genes

## Abstract

Background/Objectives: The Y chromosome plays a crucial role in male fertility, sex determination, and spermatogenesis, yet it remains poorly characterized in Mediterranean river buffalo (*Bubalus bubalis*, 2n = 50) because of its high repeat content, extensive heterochromatin, and complex palindromic structures. Although a chromosome-level Y assembly is available for swamp buffalo (2n = 48), no equivalent reference exists for the river type. Methods: To address this gap, Y chromosomes from 10 Mediterranean buffalo bulls were isolated by laser microdissection following peripheral blood culture and whole-chromosome amplification. Probe specificity was verified by FISH, and amplified Y chromosomes were sequenced using Illumina NovaSeq 6000 (Illumina, San Diego, CA, USA). Sequencing data were assembled and analysed through de novo assembly, repeat identification, sequence alignment, and variant detection. Comparative analyses included alignment to the swamp buffalo Y chromosome and annotation of Y-linked genes using the *Bos taurus* reference genome. Results: FISH confirmed the specificity of the isolated material, showing strong signals on the Y chromosome and on X/Y PAR and heterochromatic regions. Sequencing generated over 240 million paired-end reads, and de novo assembly produced 566,815 contigs. Repeat analysis identified 3.91% repetitive elements, mainly SINEs, while variant calling detected more than 23,000 variants. Comparative analyses mapped several contigs to the swamp buffalo Y chromosome and Y-linked genes. Annotation against the *B. taurus* genome identified 26 unique genes, including homologs shared with the X chromosome, and revealed MSY gene duplications, including 10 copies of *TSPY* and 3 of *HSFY*. Conclusions: These findings show that laser microdissection with NGS enables effective access to the buffalo Y chromosome, representing a milestone in the characterization of the river type genome, and providing a basis for studies on buffalo male fertility and breeding programs.

## 1. Introduction

The Mediterranean river buffalo (*B. bubalis*) represents a livestock species of major economic and biological importance, particularly in Southern Europe and Asia. In Italy, the Mediterranean buffalo is reared primarily for milk production that is almost completely processed into *Mozzarella di Bufala Campana PDO* (Protected Designation of Origin). Therefore, research and selective breeding programs have increasingly focused on milk productivity and quality traits [1,2,3,4], while relegating other quantitative traits, including fertility, and related parameters, to secondary importance.

As in humans, in domestic animals the Y chromosome also plays a pivotal role in male sex determination, spermatogenesis, and fertility, harbouring genes essential for testis development and male reproductive function [5]. However, the Y chromosome remains one of the least characterized components of the genome. Its peculiar evolutionary history, marked by progressive degeneration, accumulation of repetitive sequences, palindromic structures, and extensive heterochromatin, has made its sequencing and accurate assembly particularly challenging [6,7].

A fundamental obstacle in Y chromosome sequencing arises from its lack of a homologous counterpart across most of its length. Except for the pseudoautosomal regions (PARs), which recombine with the X chromosome during male meiosis, the male-specific region of the Y chromosome (MSY) is haploid and does not undergo recombination. Consequently, when whole-genome sequencing approaches based on genomic DNA are applied to male individuals, Y-linked sequences are typically identified by subtraction or comparative coverage against female genomes or X chromosome assemblies. This indirect strategy often leads to fragmented assemblies, collapsed repeats, misassemblies, and incomplete representation of Y-linked genes [8,9].

In bovids, these limitations are particularly evident. While high-quality reference genomes are now available for cattle, the Y chromosome remains poorly resolved, with many assemblies either excluding it entirely or representing it only partially. In the water buffalo, genomic studies have primarily focused on autosomes and the X chromosome, whereas the Y chromosome has received limited attention. Most existing knowledge on the buffalo Y chromosome derives from cytogenetic studies and marker-based analyses [5,10,11,12]. Only recently high-resolution sequencing efforts have been made to generate the most detailed sequence-level information on the buffalo Y chromosome for the swamp buffalo (*B. bubalis carabanensis*) [13].

Studies combining comparative genomics and targeted sequencing have identified conserved Y-linked genes shared with cattle, such as *SRY* (Sex-determining Region Y), *ZFY* (Zinc Finger Y) and *UTY* (Ubiquitously transcribed tetratricopeptide Y), and highlighted structural similarities between bovid Y chromosomes [14]. However, these investigations relied largely on short-read sequencing of whole genomic DNA and comparative alignment to cattle Y references, resulting in fragmented contigs and limited resolution of repetitive and ampliconic regions. Importantly, equivalent high-resolution data are still lacking for the Mediterranean river buffalo, despite its distinct evolutionary history and breeding context.

Recent advances in long-read sequencing and chromosome-scale assemblies have significantly improved autosomal and X chromosome reconstruction in livestock species. Nevertheless, even with third-generation sequencing technologies, the Y chromosome remains problematic due to its low complexity and high repeat content when sequenced as part of whole-genome libraries [15]. This has prompted renewed interest in alternative strategies aimed at physically isolating chromosomes prior to sequencing.

In this context, laser capture microdissection (LCM) represents a powerful yet underutilized approach for the study of complex genomic regions. By enabling the direct isolation of individual chromosomes from metaphase spreads, LCM circumvents the confounding presence of homologous chromosomes and autosomal DNA. This strategy has previously been applied with success to human and animal chromosomes, allowing targeted sequencing of structurally complex or repetitive genomic regions [16,17].

Here, we present a high-throughput sequencing approach based on the direct isolation of the Y chromosome by laser microdissection in the Mediterranean river buffalo. By combining chromosome-specific microdissection with next-generation sequencing (NGS), we generated a Y-enriched sequencing dataset that minimizes X-derived contamination and improves assembly accuracy of male-specific regions. This approach provides a robust framework for overcoming long-standing technical barriers in Y chromosome genomics and offers new opportunities for investigating male fertility, Y chromosome evolution, and species-specific genomic features in *B. bubalis*.

## 2. Materials and Methods

### 2.1. Ethics Statement

All experimental procedures involving animals were conducted in accordance with the European Union Directive 2010/63/EU on the protection of animals used for scientific purposes and complied with national and institutional guidelines for animal care and use. Peripheral blood samples were collected from clinically healthy male Mediterranean river buffalo (*B. bubalis*) during routine veterinary procedures, with no additional discomfort or harm to the animals. The study was approved by the Bioethics Committee of the University of Torino (Prot. N. 0239749 on 17 May 2022).

### 2.2. Lymphocyte Cell Cultures and Laser Microdissection

Lymphocyte cell cultures from ten buffalo bulls were prepared according to the standard cytogenetic techniques [18]. Briefly, whole blood was cultured in RPMI 1640 medium supplemented with Foetal Bovine Serum (10% *v*/*v*), L-glutamine (30 mg/mL), antibiotics-antimycotic solution (0.5×), and Concanavalin A (1mg/mL) to stimulate lymphocyte proliferation. Cultures were incubated at 37 °C for 72 h. Three replicates for each sample were prepared, and at the harvesting, all replicates were subjected to 50 min of colcemid treatment (0.05 μg/mL) to arrest the mitotic spindle and block the metaphases, followed by hypotonic treatment in 0.075 M KCl and fixation in methanol:glacial acetic acid (3:1, *v*/*v*). Fixed cell suspensions were dropped onto clean glass slides and air-dried. Slides were examined under phase-contrast microscopy for quality check. Replicates with well-spread metaphases were then selected for laser capture microdissection. Before that, the buffaloes were karyotyped and were all karyologically normal.

For laser microdissection, the fixed lymphocyte suspension was then spread onto a polyethylene naphthalene membrane attached to a thin glass slide (PALM Microlaser system, Carl Zeiss Micro Imaging GmbH, Jena, Germany), allowed to dry, and GTG-banded followed Seabright [19]. The Y chromosome was identified based on its characteristic size, morphology and banding in the buffalo karyotype and was selectively isolated according to Kubickova et al. [20] using a PALM MicroLaser system (Carl Zeiss Micro Imaging GmbH, Jena, Germany) coupled with an inverted microscope under an oil immersion objective (100× magnification). Microdissected chromosomes were catapulted by a single laser pulse and were collected into sterile microcentrifuge PCR caps containing 4 μL of PCR oil. At least 10 copies of the Y chromosome were microdissected from metaphase spreads of each individual buffalo bull to ensure sufficient DNA yield and to minimize stochastic amplification bias.

### 2.3. Whole Chromosome Amplification

Whole-chromosome amplification of the microdissected material was primarily achieved using a Degenerate Oligonucleotide Primer (DOP) PCR amplification in a reaction volume of 15 μL containing 0.75U Taq DNA polymerase (Invitrogen Life Technologies, Carlsbad, CA, USA), following the conditions described by Kubickova et al. [20] with an initial denaturation step at 96 °C for 3 min followed by 8 cycles at 96 °C for 1 min, 30 °C for 1 min with 2 min of transition from 30 °C to 72 °C, and 72 °C for 2 min. Amplification carried on at 94 °C for 1 min, 56 °C for 1 min and 72 °C for 2 min with a final extension of 5 min at 72 °C. To increase the DNA yield, a second DOP-PCR was performed using 5 μL of the primary PCR as template in a total volume of 50 μL. An initial denaturation step at 95 °C for 3 min was performed, followed by 30 cycles at 94 °C for 15 s, 56 °C for 30 s, 72 °C for 2 min and a final extension at 72 °C for 5 min. The amplified material obtained from this second reaction was purified using MinElute PCR purification kit (Qiagen, Hilden, Germany) and diluted to obtain a final concentration of 50 ng/μL.

### 2.4. Probes Preparation and Fluorescent In Situ Hybridization (FISH)

Fluorescence in situ hybridization (FISH) was carried out as a validation step to confirm the specificity of the microdissected Y chromosome material before the sequencing for each bull. For this reason, a DOP-PCR using 2 μL of products from the first DOP-PCR reaction was labelled in a separate reaction using the Spectrum Orange-dUTP (Abbott Molecular, Des Plaines, IL, USA) using the protocol reported by Pauciullo et al. [21]. Similarly, all steps of the FISH procedure, including probe precipitation, denaturation of probes and chromosome preparations, hybridization, and post-hybridization washes, were performed following the protocol described by Pauciullo et al. [21].

### 2.5. Library Construction and High Throughput Sequencing

Sequencing libraries were prepared using the NEBNext^®^ Ultra™ II DNA Library Prep Kit for Illumina (New England Biolabs, Ipswich, MA, USA), a ligation-based workflow optimized for low-input and amplified DNA. Library construction was performed according to the manufacturer’s instructions and included combined end repair and dA-tailing reactions, followed by adaptor ligation and PCR enrichment, while minimizing purification steps to preserve library complexity and yield. For each library, 50 ng of amplified DNA obtained from laser-microdissected chromosomes and quantified by Qubit fluorometric analysis (Life Technologies, Carlsbad, CA, USA) was used as input material. Following adaptor ligation, libraries were enriched using a limited number of PCR cycles to reduce amplification bias. The resulting libraries showed insert size distributions predominantly ranging from approximately 200 to 400 bp, including adaptor sequences. Library concentration, fragment size distribution, and molar concentration were assessed using Qubit fluorometry, an Agilent Bioanalyzer, and quantitative PCR with the KAPA Library Quantification Kit (KAPA Biosystems, Boston, MA, USA). Libraries were normalized to the appropriate loading concentration and sequenced on an Illumina NovaSeq 6000 platform using a paired-end configuration, generating 2 × 150 bp reads.

### 2.6. Sequencing Data Processing and Y-Chromosome Sequence Assembly

Raw sequencing data were processed prior to downstream analysis using TrimGalore (v0.6.7) to remove adapter sequences and perform quality trimming, applying a Phred quality threshold of 30. The trimmed reads were subsequently aligned against the 16S rRNA reference using the BWA aligner (v0.7.17-r1188) in order to remove potential bacterial contamination. Reads that did not map to the 16S rRNA reference were retained and used for de novo assembly of Y chromosome sequences.

De novo assembly was performed using SPAdes v3.15.5 with default parameters. The assembly workflow included automatic read error correction and repeat resolution. Assembly quality and contiguity statistics were evaluated using QUAST (V5.2.0) with default parameters. Contigs shorter than 500 bp were excluded from further analyses.

Repetitive elements within the assembled contigs were identified using RepeatMasker with default parameters. Microsatellite motifs were detected using MISA (v2.1).

### 2.7. Read Alignment and Variant Detection

Trimmed reads were aligned to the assembled contigs using the BWA aligner (v0.7.17 -r1188). The resulting alignments were converted to BAM format, sorted, and indexed using SAMtools (v1.18) and PCR duplicates were removed prior to variant detection. Alignment statistics and sequencing depth were also calculated using SAMtools.

Variant calling was performed using the Genome Analysis Toolkit (GATK) pipeline (v4.4.0.0), with the HaplotypeCaller tool (v4.4.0.0). Deduplicated BAM files from the ten sequenced samples were analysed jointly to generate a multi-sample VCF file containing all detected variants. Variant calling was performed using diploid settings implemented in GATK. No additional filtering thresholds were applied in order to retain potentially informative variants.

### 2.8. Comparative Genomic Analysis and Identification of Y-Linked Gene Variants

To characterize the genomic origin of the assembled contigs, they were aligned to the swamp buffalo Y-chromosome sequence available in Figshare [13] to identify Y-linked genomic regions. Sequence similarity searches were performed using the BLASTn algorithm, and hits with sequence identity ≥ 80% and E-values ≤ 1 × 10^−5^ were retained.

Gene coordinates provided in the corresponding GFF annotation file were used to extract Y-linked gene sequences from the swamp buffalo Y-chromosome assembly using Integrative Genomics Viewer (IGV). The extracted gene sequences were further validated by comparison with the NCBI reference database. Specifically, the sequences were aligned against the *B. taurus* RefSeq genome (ARS-UCD2.0) in order to identify unique gene entries and remove redundant sequences as well as flanking genomic regions. The resulting set of unique gene sequences was then used as a reference to identify homologous regions within the assembled contigs using BLASTn. Contigs showing significant similarity (sequence identity ≥ 80% and e-value ≤ 1 × 10^−5^) to annotated Y-linked genes were retained. Genomic coordinates of the aligned contigs were inferred using the alignment positions obtained from BLAST together with the genomic coordinates of the corresponding genes. Variants detected in contigs were subsequently projected onto genomic coordinates and intersected with gene and exon annotations in order to identify variants located within coding regions.

**Figure 1:**
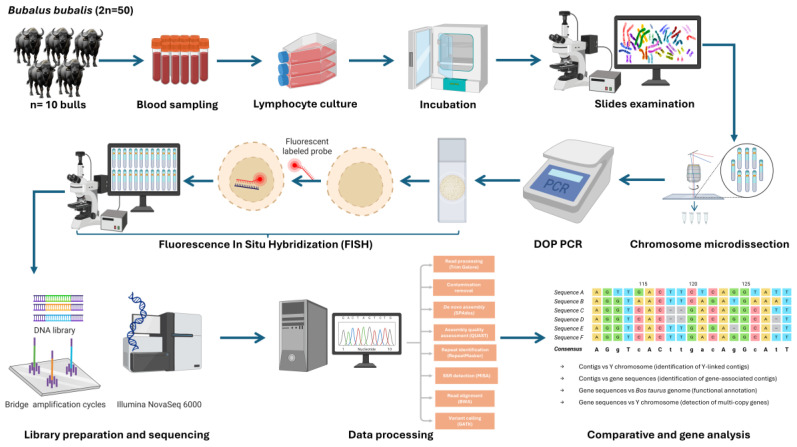
Workflow of Y chromosome sequencing and characterization in Mediterranean river buffalo. Schematic overview of the experimental and bioinformatics workflow applied in this study. The workflow includes blood sampling and cell culture, Y chromosome isolation by laser capture microdissection, whole-chromosome amplification using DOP-PCR, validation by FISH, Illumina sequencing and downstream data processing including quality control, de novo assembly, repeat identification and variant calling. Comparative and gene analyses were subsequently performed to identify Y-linked contigs, annotate gene sequences and assess gene copy number variation.

To investigate potential gene duplication events on the Y chromosome, the gene sequences were also aligned against the swamp buffalo Y chromosome using the BLASTn algorithm. High-confidence hits were identified using a minimum sequence identity threshold of ≥95% and an E-value cutoff of ≤1 × 10^−5^. The genomic coordinates of significant hits were examined to identify genes present in multiple copies, and redundant hits mapping to the same genomic *loci* were collapsed to retain only unique positions. A schematic overview of the experimental design and downstream bioinformatics workflow is provided in Figure 1.

## 3. Results

**Figure 2:**
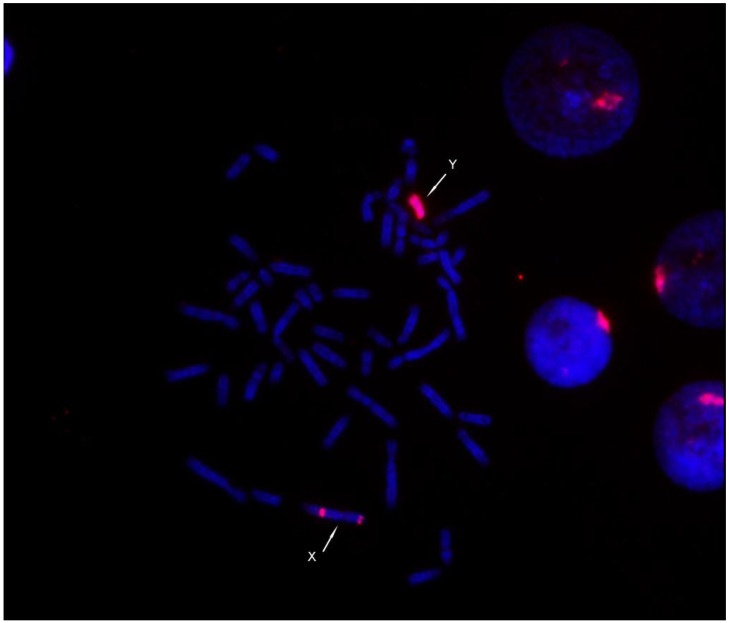
FISH showing the specific painting signal of Y-chromosome and dot signals both for the PAR and the heterochromatic block of the X-chromosome on the metaphase and interphase nuclei.

The chromosome-specific painting probes were tested on metaphase chromosome preparations of river buffalo (2n = 50, XY). For each of the ten individual probes produced, the fluorescent in situ hybridisation generated intense and specific signals on the whole Y and two signals on the X chromosome corresponding to the PAR and a heterochromatic region, respectively (Figure 2). Specific signals were visible also in the interphase nuclei and no specific signals were detected on autosomal chromosomes. Based on these results, validation was considered as achieved and the probes were subsequently used for the high throughput sequencing.

### 3.1. Sequencing Data Processing and Y-Chromosome Sequence Assembly

A total of 240,243,862 paired-end Illumina reads (120,121,931 read pairs) were generated across the ten sequenced samples. The mean read length ranged from 150 to 151 bp, and the GC content varied between 46% and 54%. After quality trimming, an average read length of 147–149 bp was retained, and 0.9–4.4% of reads were removed per sample. De novo assembly of the trimmed reads produced 566,815 contigs with a total length of 57,474,490 bp. Among these, 3254 contigs were ≥500 bp, spanning 2,260,027 bp, while 255 contigs were ≥1000 bp, covering 292,844 bp. The largest contig had a length of 1578 bp. The GC content of the assembly was 48.11%. Assembly contiguity statistics showed an N50 of 689, N 90 of 529, L 50 of 1,292 and L90 of 2,814. No ambiguous bases (Ns) were detected per 100 kbp (Table 1).

RepeatMasker analysis revealed the presence of repetitive elements across the assembled contigs, accounting for 3.91% of the total sequence length. Interspersed repeats were dominated by short interspersed elements (SINEs), which represented the most abundant class (*n* = 436), whereas long interspersed elements (LINEs) were detected at low frequency (*n* = 6). Additional repeat categories included satellite sequences (*n* = 14), ribosomal RNA fragments (*n* = 7), transfer RNAs (*n* = 5) and small nuclear RNAs (*n* = 4), as well as simple repeats and low-complexity regions (Table S1). Microsatellite analysis using MISA identified a total of 549 simple sequence repeats (SSRs) across the assembled contigs, including 326 mononucleotide repeats and 127 di-, tri-, tetra- and penta-nucleotide microsatellites. Repeat lengths ranged from 10 to 80 bp, with the longest motif corresponding to a pentanucleotide repeat (TGTGG)_16_. Additionally, 96 compound repeat regions were detected. A detailed list of identified SSR is provided in Table S2.

### 3.2. Alignment and Variant Detection

Variant calling across the ten samples identified 23,418 variants, including single nucleotide polymorphisms (SNPs), insertion/deletion variants (indels) and multi-allelic sites. The number of variants detected per sample ranged from 956 in sample 8 to 12,459 for sample 2. In total, 3719 insertions, 4413 deletions, 968 multi-allelic SNPs and 14,318 SNPs were identified.

### 3.3. Comparative Genomic Analysis and Identification of Y-Linked Gene Variants

**Figure 3:**
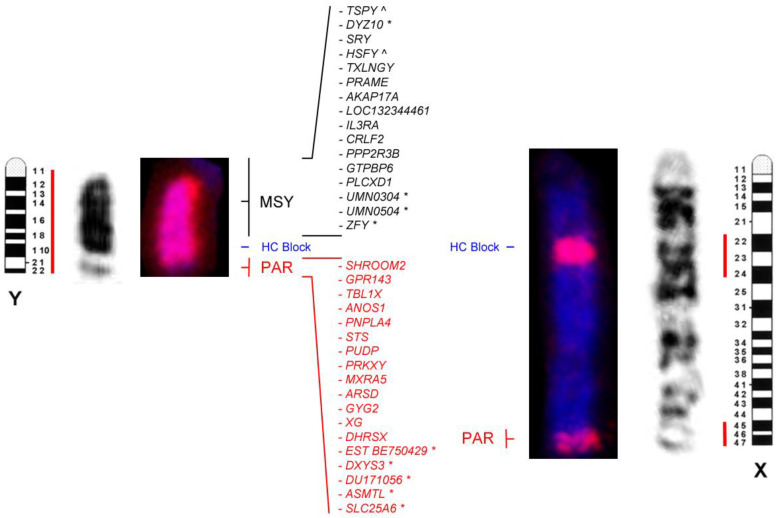
Structural organization of the river buffalo sex chromosomes and chromosomal localization of Y-linked and pseudoautosomal genes. Representative G-banded ideograms and chromosome images of the Y chromosome (**left**) and X chromosome (**right**) are shown together with FISH signals identifying the male-specific region of the Y chromosome (MSY), the heterochromatic (HC) block, and the pseudoautosomal region (PAR). Genes identified within the MSY and PAR are listed in the central panel according to their chromosomal order. However, the arrangement of genes does not reflect their actual physical distances along the chromosome. Carets indicate multi-copy genes, whereas asterisks denote genes physically mapped by FISH by other authors (see Rossetti et al. [5] for reference).

BLAST analysis of the 3254 contigs ≥ 500 bp against the swamp buffalo Y chromosome produced 20,217 alignments, indicating that several contigs mapped to multiple genomic locations. Of these, 18,600 alignments met the filtering criteria of ≥80% and e-value ≤ 1 × 10^−5^ (Table S3). Alignment of all assembled contigs against the set of Y-linked genes, produced 37,995 significant hits, showing that multiple contigs matched known Y-linked gene sequences (Table S4). BLASTing our contigs with the entries reported by Wang et al. [13] against the *B. taurus* reference genome we were able to identify 26 unique genes (Figure 3) among the 52 originally reported in the swamp buffalo [13].

We found that among these entries, three corresponded to autosomal *loci* located on chromosomes 5, 11 and 20, while other entries represented duplicated annotations, like the pseudouridine-5′-phosphatase (*PUDP*) and the glycogenin 2 (*GYG2*). Among the 26 unique genes retained for downstream analyses, 13 showed comparable BLAST similarity to both the X and Y chromosomes (Table S5). Alignment of these genes against the Y chromosome sequence revealed that several genes were present in multiple genomic locations. For instance, the analysis of multi-copy genes highlighted that in the MSY region for *TSPY1* repeated 10 times and *HSFY* repeated 3 times (Table 2). Detailed BLAST alignment statistics for the identified gene copies are provided in Table S6.

Finally, variants detected in the assembled contigs were intersected with gene annotations, leading to the identification of two variants located within coding exons of annotated Y-linked genes. One variant occurred in exon 7 of the anosmin-1 gene (*ANOS1*), while the second was identified in exon 3 of the glycogenin-2 gene (*GYG2*).

## 4. Discussion

The present study provides, to our knowledge, the first Y-chromosome-enriched sequence resource obtained by direct laser microdissection in the Mediterranean river buffalo. A particularly strong point of the workflow is the cytogenetic validation step. In fact, the painting probes hybridised along the whole buffalo Y chromosome and, as expected, also produced discrete signals on the X chromosome at the pseudoautosomal region and at the heterochromatic block, with no autosomal labelling. This pattern strongly suggests that the amplified material was exclusively obtained from the Y-gonosomal DNA and that the X signals reflected biologically shared sequence rather than random contamination. Furthermore, the absence of autosomal hybridisation signals supports the specificity of the microdissected material and may also suggest a limited abundance of repetitive elements shared between the river buffalo Y chromosome and the autosomal genome.

The combined application of laser microdissection of whole chromosomes, chromosome arms, or specific regions, together with NGS, has already been demonstrated to be a valuable strategy for improving and validating genetic maps and sequence assemblies. For instance, this approach has been successfully applied to the short arm of chromosome 7p in *Xenopus tropicalis* [16], as well as to address specific issues such as population-specific or tumor-associated rearrangements and to characterize previously unsequenced genomic regions, including centromeres [17]. Beyond its value for improving and validating assemblies, this strategy is particularly informative for sex chromosomes, where FISH validation of microdissected material can simultaneously confirm probe specificity and reveal the distribution of sequences shared between X and Y.

In bovids, the shared Y/X hybridisation is consistent with the known organisation of the pseudoautosomal domain and with the distinctive heterochromatic remodelling of the buffalo sex chromosomes. The Y-derived painting probe generated in the present study confirmed the location of the river buffalo PAR in the Xq44–47 region, as previously reported by Hassanane et al. [22] and subsequently confirmed by physical gene mapping [10,11,12]. However, this clear cytogenetic validation of Y-chromosome enrichment does not necessarily translate into continuity at the sequence level. Consistent with this, the highly fragmented de novo assembly indicates that physical enrichment of the chromosome does not, by itself, solve the long-standing problem of Y-chromosome reconstruction. The recovery of only short contigs, despite deep sequencing, is in line with the repeat-rich and multicopy architecture of mammalian Y chromosomes and is likely to reflect the combined effects of DOP-PCR amplification bias, short-read sequencing, and the abundance of low-complexity and ampliconic sequence. Therefore, the present dataset is best interpreted as a targeted Y-enriched sequence collection rather than as a near-complete assembly. Even so, it fills an important gap for Mediterranean river buffalo, whose Y chromosome remains much less resolved than the recently published male buffalo or telomere-to-telomere bovid Y references [13,14,23]. This is also confirmed by the number of multi-copy genes found in buffalo (10 for *TSPY1* and 3 for *HSFY*) similarly to what has been observed in cattle and yak for the multi-copy genes *PRAMEY*, *HSFY*, *TSPY*, *ZNF280AY* and *ZNF280BY* [14,24].

The comparative gene analysis also provides an important biological message. The reduction of the initial swamp-buffalo-derived annotation from 52 entries to 26 unique genes, together with the observation that 13 of them showed comparable similarity to both X and Y chromosomes, highlights how easily Y-linked gene catalogues can be inflated by duplicated annotations, pseudoautosomal *loci* and X-Y gametologs. The number of genes we discovered is substantially lower than those annotated in swamp buffalo [13] and cattle [14]. In the latter investigation, the annotation strategy adopted for the Angus Y chromosome, which relied on cross-species orthologs and a relatively permissive 75% identity threshold, may have overestimated the number of unique Y-linked genes by retaining *loci* with substantial homology to shared X-Y or pseudoautosomal sequences. In contrast, our approach gave results that support a more conservative gene content, more in line with the human Y chromosome [25].

The coexistence in Figure 3 of canonical MSY genes such as *SRY*, *ZFY*, *TSPY1* and *HSFY1/HSFY2* with genes that clearly belong to the shared gonosomal compartment is informative, because it indicates that the present Y-enriched dataset sampled both male-specific sequence and regions of persistent X-Y homology. This mixed signal is not unexpected in ruminants, where the PAR and adjacent X-degenerate segments have proven difficult to delimit precisely without high-quality male assemblies [13,14,23,26]. Furthermore, the present data are useful for correcting systematic misassignments that may arise in comparative-genomics-based reconstruction of heterogametic sex chromosomes. For instance, *SHROOM2* has recently been described as an X-specific gene outside the pseudoautosomal region (PAR) in domestic ruminants, including river buffalo. This organization is consistent with that reported in humans and cattle [14], where *SHROOM2* is located distal to the pseudoautosomal boundary, but differs from the situation in dogs, where the PAR extends to include *SHROOM2* [27]. Similarly to dogs, our Y-chromosome-specific sequencing approach clearly supports the presence of *SHROOM2* on the buffalo Y chromosome, thereby indicating that its genomic assignment in this species requires reconsideration.

From a functional perspective, the identification of coding variants in *ANOS1* and *GYG2* deserves attention, but also careful restraint in interpretation. *ANOS1* is of particular interest because variation in its human orthologue is implicated in congenital hypogonadotropic hypogonadism and Kallmann syndrome, linking the gene to reproductive development [28]. However, in the present study both candidate exonic variants were identified within a highly repetitive sequence space and within a gene inventory that includes shared X/Y components. Notably, *ANOS1* and *GYG2* are established pseudoautosomal genes in several mammalian species, including cattle, and their assignment to the shared gonosomal portion of the current annotation suggests that they may also reside within the PAR in river buffalo. The presence of exonic variants in these genes is therefore not unexpected, as pseudoautosomal regions are generally characterized by elevated genetic diversity resulting from ongoing X-Y recombination, larger effective population size, and increased local mutation rates associated with recombination activity [29]. In addition, variant discovery was performed jointly under diploid settings on a chromosome that is largely haploid. For these reasons, the detected coding changes should presently be considered candidate polymorphisms that require locus-specific validation and are more appropriately interpreted as reflecting heterozygosity between homologous X- and Y-linked PAR sequences rather than variation restricted to the haploid MSY [13,14,23].

## 5. Conclusions

This work provides, to our knowledge, the first sequence-based characterization of the Mediterranean river buffalo Y chromosome, enabling the identification of male-specific Y (MSY) and pseudoautosomal (PAR) gene content and establishing a foundation for future comparative and functional studies of buffalo sex chromosome evolution. Overall, the study demonstrates that laser microdissection coupled with NGS is a practical route for accessing the buffalo Y chromosome and for generating species-specific sequence resources that would be difficult to obtain from conventional whole-genome data alone. Its main strength lies not in producing a finished chromosome assembly, but in providing a proof-of-principle enrichment strategy and a first sequence framework for marker development, refinement of buffalo MSY/PAR annotation, and future studies of paternal lineages and male reproductive biology. The logical next step will be to combine chromosome-targeted enrichment with long-read sequencing (T2T approach) and chromosome-scale scaffolding so that the repetitive and multi-copy architecture of the Mediterranean river buffalo Y chromosome can be resolved with much greater continuity and compared directly with emerging high-quality bovid Y assemblies.

## Figures and Tables

**Table 1:** Summary of sequencing and assembly statistics of river buffalo Y chromosome contigs.

Metric	Value
**Sequencing metrics**	
Total reads (paired end)	240,243,862
Read pairs	120,121,931
Mean read length (bp)	150–151
GC content (%)	46–54
Post-trimming read length (bp)	149
Reads removed (%)	0.9–4.4
**Assembly metrics**	
Total contigs	566,815
Total assembly length (bp)	57,474,490
Contigs ≥ 500 bp	3254
Length of contigs ≥ 500 bp (bp)	2,260,027
Contigs ≥ 1000 bp	255
Length of contigs ≥ 1000 bp (bp)	292,844
Largest contig (bp)	1578
GC content (%)	48.11
N50 (bp)	689
N90 (bp)	529
L50	1292
L90	2814

**Table 2:** Multi-copy Y-linked genes identified in Y buffalo chromosome.

Gene Symbol	Copy ID	Gene Start	Gene End	Gene Length (bp)
***TSPY* ^1^**	1	51,262	53,230	1968
	2	67,058	69,009	1951
	3	82,844	84,796	1952
	4	98,630	100,575	1945
	5	114,400	116,348	1948
	6	130,242	132,197	1955
	7	146,061	148,034	1973
	8	3,949,688	3,951,649	1961
	9	3,965,506	3,967,471	1965
	10	4,063,227	4,065,196	1969
***HSFY* ^2^**	1	2,712,294	2,713,966	1672
	2	5,783,183	5,784,856	1673
	3	6,006,015	6,007,687	1672

^1^ *TSPY*: Testis-specific Y-encoded protein 3-like. ^2^ *HSFY*: Heat shock transcription factor, Y-linked-like.

## Data Availability

Sequence data are available from the BioProject Accession PRJNA1467996. Information on biosamples is available from SUB16198387, whereas the sequence read archive is available from the SRA accession SUB16198405.

## Supplementary Materials

The following supporting information can be downloaded at: https://www.mdpi.com/article/10.3390/genes17070740/s1, Table S1: Summary of repetitive elements identified by RepeatMasker in assembled contigs; Table S2: List of the identified simple sequence repeats (SSR); Table S3: BLASTN alignments of Y-linked contigs against the swamp buffalo Y chromosome (filtered at ≥80% identity and E-value < 1 × 10^−5^); Table S4: BLAST alignment results of assembled contigs against Y-linked genes; Table S5: Genes and intergenic regions identified from buffalo Y-derived sequences based on similarity to B. taurus reference genome.; Table S6: BLAST alignment statistics for identified Y-linked gene copies.

## Abbreviations

The following abbreviations are used in this manuscript:

NGS	Next-generation sequencing
PDO	Protected Designation of Origin
PAR	Pseudoautosomal region
MSY	Male-specific region of the Y chromosome
LCM	Laser capture microdissection
PALM	Polyethylene naphthalene membrane
DOP	Degenerate oligonucleotide primer
FISH	Fluorescence in situ hybridization
